# 

**Published:** 1901-11-30

**Authors:** 


NOTICE TO CORRESPONDENTS.

All MSS., Letters, Books for Review, and other matters intended for the Editor should be addressed The Editor, "The Hospital," The Hospital Building, 28 & 29 Southampton Street, Strand, W.C.

The Editor cannot undertake to return rejected MS., even when accompanied by stamped directed envelope.

**Notice to Advertisers and Subscribers.**

All Advertisements, Orders for Copies of The Hospital, and Business Communications should be addressed to **THE MANAGER (Not to the Editor),** The Hospital, 28 & 29 Southampton Street, Strand, London, W.C.

The Cost of Subscription, post free, is as follows:—Per week, 2½d.; per quarter, 2s. 8d.; per half-year, 5s. 3d.; per annum, 10s. 6d. Foreign:—Per annum, 15s. 2d., payable, in all cases, in advance.

**NOTICE.—Vol. XXX. of "The Hospital" (April 1901, to September 1901), in handsome cloth binding can now be obtained at the office of this Journal, price, 7s. 6d. Cases for binding the Numbers contained in this Volume 1s. 6d. Index to Vol. XXX., 2½d., post free.**

**The Monthly Part for December will be ready on December 2nd. Price 6d., by post 8d.**

### BOOKS RECEIVED.

WALTER SCOTT.

"The Criminal." By Havelock Ellis.

"The Evolution of Sex." By Professor Patrick Geddes and J. Arthur Thomson.

"Manuals of Employment for Educated Women." No. IV., Medicine. By Christabel Osborn. With an introduction by Mrs. Garrett Anderson, M.D.

J. B. LIPPINCOTT AND CO.

"Photographic Atlas of the Diseases of the Skin." By George Henry Fox, A.M., M.D.

LEA BROTHERS, PHILADELPHIA.

"The History of the Development of Medical Science in America." By H. R. M. Landis, M.D.

CASSELL AND CO.

"The Practical Nursing of Infants and Children." By Frank Cole Madden, M.B., B.S.Melb., F.R.C.S.

(even in cases of supposed "ordinary cold"), the absolute necessity of the proper ventilation of sleeping rooms, and other simple rules of hygiene.

### Scraps and Gleanings.

The King has presented twenty pheasants for the inmates of the Cancer Hospital, Fulham Road, S.W.

The British and Foreign Sailors' Society have received for its Passmore Edwards Sailors Home £500 from Mr. James Coats, jun.; £50 from Mrs. Cunningham to endow a bed; and £50 from Mrs. Cadbury, to endow a "Richard Cadbury" bed.

Out of Islington's population of 340,000, of whom 41,000 are householders, only 444 persons were subscribers to the Great Northern Central Hospital during the year, while the regular income that the hospital could depend upon was only £5,000, as against an expenditure of £13,000.

A sum of £50,000 is required for the new Belgrave Hospital for Children, £15,000 of which has been collected, and a further sum of £5,000 is promised, providing £5,000 has been collected by December next in sums of not less than £100. The site of the new hospital is suitable to accommodate 80 beds, but the building is to be built block by block in order to avoid initial debt, and is to be commenced at once. Among the new features of the building is to be a Baby Ward for the training of nursemaids in the care of infants.

The chairman of the committee of the Shipwrecked Mariners' Society makes a special appeal for funds in aid of this charity, which provides for the care of shipwrecked persons, relieves the widows and orphans of sailors and fishermen, and encourages thrift among the seafaring classes. Since 1839 considerably more than half a million persons have been so assisted.

### The Student of Medicine.

### Appointments.

**J. A. Corbitt**, M.B., B.Ch., B.A.O.R.U.I., appointed Certifying Factory Surgeon for the Bangor District of County Down. **A. Forsyth**, M.B., C.M., appointed District Medical Officer of the Truro Union. **J. C. Padwick**, M.R.C.S., L.R.C.P.Lond., appointed Certifying Factory Surgeon for the Borough and Rural District of Bridgnorth. **Leonard A. Parry**, F.R.C.S.Eng., B.S., M.D.Lond., appointed Assistant Surgeon to the Sussex Eye Hospital, Brighton. **M. S. Paterson**, M.B., B.S.Durh, M.R.C.S., L.R.C.P., appointed Resident Medical Officer to the Hospital for Consumption and Diseases of the Chest, Brompton. **F. E. Taylor**, M.A., M.B., Ch.B., M.Sc., M.R.C.S., L.R.C.P., late Resident Medical Officer, appointed Registrar to the Chelsea Hospital for Women **George Thornton**, M.D., M.S.Edin., M.R.C.P.Lond., M.R.C.S.Eng., D.P.H.Oxford, appointed Medical Superintendent of the Civil Hospital, Pretoria. **Redmond Roche**, A.B., M.R.C.S.Eng., L.R.C.P.Lond, L.M., appointed Attending Medical Officer, Western Dispensary, Westminster.

